# Interleukin-17 expression in murine pressure ulcer tissues

**DOI:** 10.3892/etm.2013.912

**Published:** 2013-01-21

**Authors:** WEI CUI, LEI-FANG YANG, WEN-HONG WEI, YA-QIN ZHU, XIAO WU, PEI-XIA MU, SHU-PING GUO

**Affiliations:** 1 Departments of Special Medical Service, The Central Hospital of Xinxiang, Xinxiang, Henan 453600, P.R. China; 2 Cardiology, The Central Hospital of Xinxiang, Xinxiang, Henan 453600, P.R. China; 3 Surgical Oncology, The Central Hospital of Xinxiang, Xinxiang, Henan 453600, P.R. China

**Keywords:** pressure ulcer, animal model, interleukin-17

## Abstract

To explore the process of pressure ulcer formation, interleukin (IL)-17 expression levels were observed in a mouse model of pressure ulcers. Twenty mice were divided into experimental and control groups (10 mice per group). A mouse model of pressure ulcers was established by inducing ischemia-reperfusion injury on local tissue in the experimental group. Pressure ulcer tissues in the experimental group and normal mouse tissue in the control group were stained using hematoxylin and eosin (H&E) and observed using light microscopy. The protein and mRNA expression levels of IL-17, in mouse pressure ulcer tissues from the experimental group and in the normal tissue from the control group, were determined using real-time PCR and western blot analysis, respectively. The mRNA and protein expression levels of IL-17 were compared between the two groups. H&E staining indicated that striated muscle was arranged orderly and cellular structure was intact in the control group, whilst inflammatory cell infiltration was observed in the muscle tissue of the experimental group. The expression levels of IL-17 mRNA were 0.307±0.058 ng in the experimental group and 0.112±0.042 ng in the control group (P<0.05). The expression levels of the IL-17 protein were 0.434±0.097 ng in the experimental group and 0.181±0.040 ng in the control group (P<0.05). IL-17 expression levels were increased in pressure ulcers, which suggests that IL-17 may be associated with pressure ulcers.

## Introduction

Pressure ulcers, also know as bedsores, occur when long-term pressure to local tissue causes disruption to blood flow and tissue nutritional deficiency, which leads to skin ulceration and necrosis (1). Pressure ulcers have always been a difficult problem in clinical care and are one of the most common complications in bedridden patients. Patients with severe pressure ulcers may develop septicemia and this often results in mortality. Therefore, it is crucial to discover methods to prevent and treat pressure ulcers effectively. Interleukin (IL)-17 is a cytokine which is associated with inflammatory reactions (2). This study aimed to investigate the role of IL-17 in pressure ulcers to determine how they may be related. A mouse model of pressure ulcers was established and IL-17 expression was observed in an attempt to find an effective method to prevent and treat pressure ulcers.

## Materials and methods

### Animals

All methods in this study were approved by the Ethics Committee of the First Affiliated Hospital of Liaoning Medical University. Twenty 8-week-old BALB/c mice of either gender and weighing 25–28 g were purchased from the Experimental Animal Center, Dalian Medical University [License number: SCXK (Liaoning) 2008–0002].

### Apparatus and reagents

A TC-512 gene amplification instrument, a UV Analyzer, a GeneGenius automated gel imaging system, a BIO-RAD semi-dry transfer instrument, an IBox 600 *in vivo* imaging system, an RNA PCR kit (AMV) Ver 3.0 and Sepharose^®^ were purchased from Takara Bio, Inc. (Dalian, China). IL-17 and β-actin primers were synthesized by Shanghai Sangon Biological Engineering Technology and Service Co., Ltd. (Shanghai, China). IL-17 (H-132) rabbit polyclonal antibody was purchased from Santa Cruz Biotechnology, Inc. (Santa Cruz, CA, USA). Goat anti-rabbit IgG was purchased from Beijing Zhongshan Jinqiao Biotechnology Co., Ltd. (Beijing, China). Hematoxylin, 4% formaldehyde and eosin were purchased from Chemical Reagent Factory (Shanghai, China).

### Grouping

Twenty mice were divided into experimental and control groups (10 mice per group). Ischemia-reperfusion injury was induced on local tissue in the experimental group.

### Preparation of pressure ulcer mouse model

A mouse model of pressure ulcer was produced in accordance with previously described methods (3,4). Mice were fasted for 12 h prior to surgery, anesthetized by intraperitoneal injection of pentobarbital sodium (0.5 mg/10 g) and skin preparation was performed. A sterile metal magnetic disk (5×12 mm, 2.4 g, 1000 Gauss) was placed on the skin at the hip joint and another metal magnetic disk (5×12 mm, 2.4 g, 1000 Gauss) was placed at the groin to produce a magnetic force of 50 mmHg (1 mmHg, 0.133 kPa, 40.7 g). In the experimental group, 2 h of ischemia and 0.5 h of reperfusion were employed in a cycle; five cycles were performed to induce a pressure ulcer. To ensure the balance of water and electrolytes, 0.5 ml glucose-saline solution was administered via the caudal vein every 2.5 h (5). When the experiment had ended, the mice were sacrificed by cervical dislocation.

### Criteria for successful models

The gross appearance of mouse skin was red with breakages, ulceration and necrosis. Pathological changes, including muscle fiber atrophy, widened interstitial spaces, inflammatory cell infiltration and unclear transverse striation, were observed in mouse pressure ulcers under a light microscope.

### Hematoxylin and eosin (H&E) stain for mouse muscle tissue in pressure ulcer

Muscle tissue in the pressure ulcer was fixed with 4% paraformaldehyde for 12 h, dehydrated with gradient alcohol and washed twice with xylene for 0.5–1 h. Once it had become transparent, the muscle tissue was embedded in paraffin and sliced into sections.

Following deparaffinization with xylene, the sections were dehydrated with down-gradient alcohol, stained with hematoxylin for 5–10 min and washed with distilled water for 10 min. Several seconds after the addition of 1% hydrochloric acid alcohol, the sections were washed with tap water for 30–40 min, dehydrated with up-gradient alcohol, rendered transparent by immersion in xylene for 10 min and mounted with neutral gum for observation under a light microscope.

### IL-17 mRNA expression in mouse pressure ulcer tissue determined using real-time (RT)-PCR

Muscle tissue (∼50 mg) was placed in a sterile Eppendorf (EP) tube and the total RNA was extracted according to the manufacturer’s instructions. The reverse transcription of cDNA was performed according to the manufacturer’s instructions for RNA PCR kit (AMV; Takara, Dalian, China) Ver 3.0. Reverse transcription was performed in a 10 μl volume containing 1.0 μl 10X RT buffer, 2.0 μl MgCl_2_, 3.75 μl RNA-free dH_2_O, 1.0 μl dNTP mixture, 0.25 μl RNase Inhibitor, 0.5 μl AMV Reverse Transcriptase, 0.5 μl oligo(dT) adaptor primer and 1 μl total RNA. The reaction conditions were as follows: 30°C for 10 min, 42°C for 30 min and 99°C for 5 min. Samples were stored at −20°C for future use. In PCR, the primers used for IL-17 (6) were P1, 5′-AGATCTGGACGCGCAAACATGAG-3′ and P2, 5′-GGGTCGTCGACGGGTCTCTGTTTAG-3′ with an amplified fragment of 516 bp. The primers used for β-actin (7) were P1, 5′-AGAGGGAAATCGTGCGTGAC-3′ and P2, 5′-CAATAGTGATGACCTGGCCGT-3′ with an amplified fragment of 138 bp. PCR was performed in a volume of 20 μl containing 2.0 μl 10X PCR buffer (Mg^2+^-free), 1.0 μl of 25 mmol/l MgCl_2_, 1 μl of 2 mmol/l dNTP, 0.5 μl up- and downstream IL-17 primer, 0.5 μl up- and downstream β-actin primer, 2 μl cDNA template, 0.2 μl Ex *Taq* HS and 11.8 μl ultrapure water. Reaction conditions were as follows: 94°C for 5 min, 94°C for 30 sec, 51°C for 45 sec and 72°C for 30 sec for 35 cycles. Finally elongation was carried out at 72°C for 5 min. PCR products underwent 1.5% agarose gel electrophoresis. Images were obtained using a UV Analyzer. Grayscale values were obtained and analyzed with GeneGenius and GeneTool analysis systems.

### IL-17 protein expression in mouse pressure ulcer tissue determined using western blot analysis

Muscle tissue (∼50 mg) was placed in a sterile EP tube and cell disruption was performed. The protein was extracted and its concentration was determined using the bicinchoninic acid (BCA) method. Samples were prepared according to the protein concentrations and were boiled for 5 min following addition of reducing sample buffer (RSB) and Tris-buffered saline (TBS). The samples were stored at −20°C for future use. Each sample (20 μl) underwent SDS-PAGE and was then transferred to a membrane. The membrane was sealed with 1% bovine serum albumin (BSA) for 1 h, followed by the addition of anti-IL-17 polyclonal antibodies for overnight incubation. The membrane was washed with TBS for 5 min three times. Horseradish peroxidase-conjugated secondary antibody was added for a 1-h incubation. The membrane was washed with TBS for 5 min three times, followed by enhanced chemiluminescence (ECL) coloration in the dark. Grayscale values were analyzed using an IBox 600 *in vivo* imaging system (Tianmei Scientific Instrument Co., Ltd., Shanghai, China).

### Statistical analysis

Statistical analysis was performed using SPSS software. The data are expressed as mean ± SD. An independent sample t-test was used for comparisons between the two groups. P<0.05 was considered to indicate a statistically significant difference.

## Results

### Macroscopic observation

In the experimental group, mouse skin integrity was damaged and exhibited exudation, erosion and necrosis. In the control group, no marked changes were observed in mouse skin.

### Changes in mouse pressure ulcer muscle tissue viewed under a light microscope

**Figure 1.**
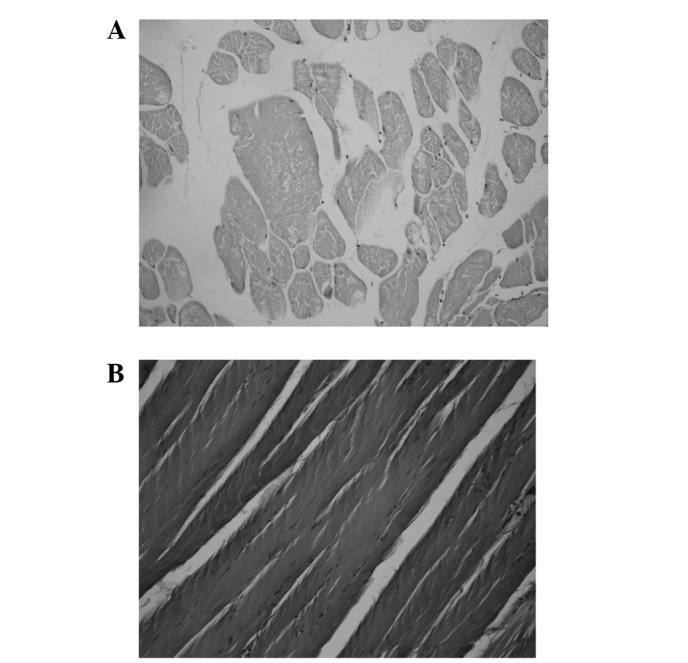
(A) H&E staining for mouse pressure ulcer muscle tissue in the experimental group. (B) H&E staining for normal muscle tissue in the control group. Magnification, ×400. H&E, hematoxylin and eosin.

In the experimental group, degenerative tearing, disappearance of transverse striation, myolysis and inflammatory cell infiltration were observed in skeletal muscle. Cell infiltration included neutrophilic granulocytes with pink cytoplasm and a blue lobulated nucleus and lymphocytes with less cytoplasm and a large nucleus (Fig. 1A). In the control group, striated muscle was arranged in order and cellular structure was intact (Fig. 1B).

### IL-17 mRNA expression in mouse pressure ulcer tissue determined using RT-PCR

**Figure 2.**
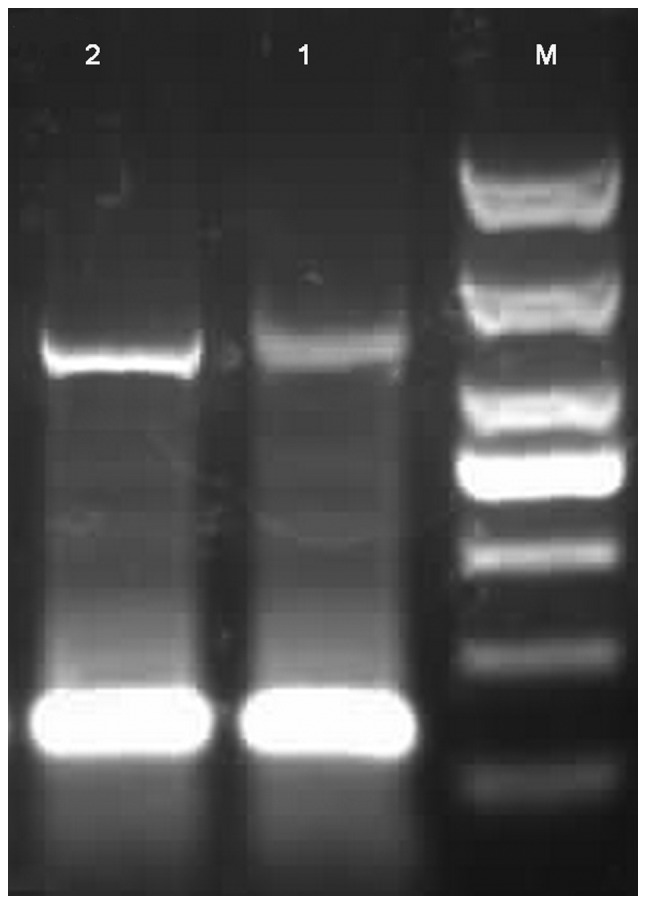
Interleukin-17 mRNA expression in mouse pressure ulcer and normal tissue. Lane M, DL1000; lane 1, control group; lane 2, experimental group.

RT-PCR showed the specific band for 516 bp in the two groups. Compared with the control group, IL-17 mRNA expression was significantly upregulated in the experimental group (P<0.01; Table I and Fig. 2).

### IL-17 protein expression in mouse pressure ulcer tissue determined using western blot analysis

**Figure 3.**
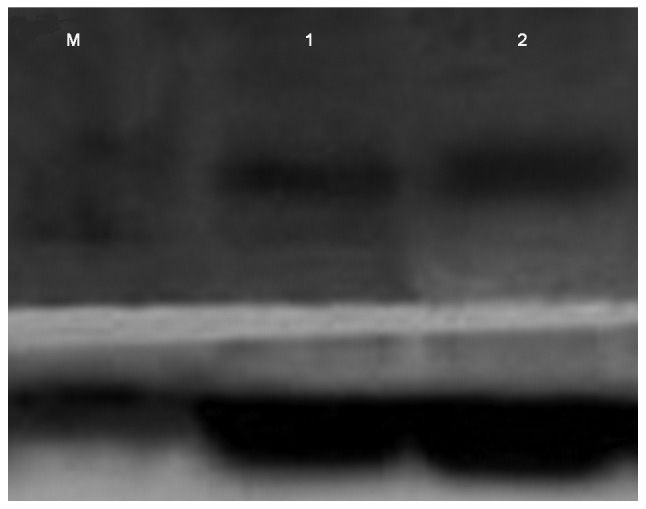
Interleukin-17 protein expression in pressure ulcer muscle tissue. Lane M, DL1000; lane 1, control group; lane 2, experimental group.

Western blot analysis showed the specific band for 15 kDa in the two groups. Compared with the control group, IL-17 protein expression was significantly upregulated in the experimental group (P<0.01; Table II and Fig. 3).

## Discussion

Long-term pressure on soft tissue causes disruption to blood circulation and vascular endothelial cell injury. Continuous platelet agglomeration leads to the occurrence of microcirculation thrombosis, which aggravates local tissue ischemia (8). Long-term tissue ischemia and hypoxia may induce metabolic disorders in tissue and cells and changes in plasma colloid osmotic pressure, which leads to cellular edema and perhaps even cell rupture (9). Damage to the tissue produces self-defensive and protective reactions to recover normal function. However, metabolic compensation is not able to maintain normal function for long, due to long-term demands on the local tissue and when metabolic disruption results in the production of oxygen free radicals and their derivatives, tissue damage is aggravated (10). This results in severe tissue damage which causes infection and inflammation to occur repeatedly. In this study, pressure ulcer mouse models were established and IL-17 expression was observed in an attempt to find an effective method to prevent and treat pressure ulcers.

H&E staining results revealed neutrophil and lymphocyte infiltration of mouse muscle tissue in the experimental group and well-arranged striated muscle and intact cells in the control group. IL-17 (IL-17A) is mainly secreted by Th17 cells of the CD4^+^ T lymphocyte subset. Th17 cells exert their biological effects via the secretion of IL-17A, IL-17F, IL-6, IL-22 and TNF-α, which signals neutrophils to move towards the site of inflammation and play an infection-fighting role in the early stage of the immune response (11–13). The results of this study indicated that the expression of IL-17 mRNA and protein occurred in the two groups, however these expression levels were significantly increased in the experimental group compared with the control group (P<0.01). This is suggestive of overexpression of IL-17 in mouse pressure ulcer muscle tissue, which is associated with the occurrence and development of inflammatory lesions. IL-17 stimulates the secretion of IL-6, TNF-α, granulocyte macrophage colony-stimulating factor (GM-CSF), IL-8, IL-6, IL-1β and G-CSF, which allows neutrophils, polymorphonuclear cells, T cells and macrophages to move to the inflammation site and carry out an immune function. In this study, H&E staining results indicated that neutrophil and lymphocyte infiltration occurs in mouse pressure ulcer muscle tissue, suggesting that IL-17 may contribute to an immune response in the development of pressure ulcers. Differentiation occurs earlier in Th17 cells than in Th1 and Th2 cells, there are fewer Th17 cells than Th1 and Th2 cells and the effective response time is shorter in Th17 cells than in Th1 and Th2 cells, therefore it is crucial to extend the effective response time and to increase the number of Th17 cells to improve the immune effects of IL-17. When the number of Th17 cells reaches a certain threshold, Th1, Th2 and Treg cells inhibit Th17 cell differentiation. Therefore, in the persistent infection of pressure ulcers, a replacement for the role of IL-17 secreted by Th17 cells is required. Studies have shown that IFN-γ is increased in the late phase of inflammation (14), however, whether IFN-5 plays a major role in severe pressure ulcers remains to be confirmed by future studies.

Pressure ulcer is a complex problem in clinical care. Further studies are required to discover better methods to prevent and treat pressure ulcer.

## Figures and Tables

**Table I.** IL-17 mRNA expression in mouse pressure ulcer muscle tissue (mean ± SD).

Group	Mice (n)	IL-17 mRNA	t	P-value
Experimental	10	0.307±0.058	8.595	0.000
Control	10	0.112±0.042		

IL-17, interleukin-17.

**Table II.** IL-17 protein expression in mouse pressure ulcer muscle tissue (mean ± SD).

Group	Mice (n)	IL-17	t	P-value
Experimental	10	0.434±0.097	7.608	0.000
Control	10	0.181±0.040		

IL-17, interleukin-17.
